# ImmunoDisk—A Fully Automated Bead-Based Immunoassay Cartridge with All Reagents Pre-Stored

**DOI:** 10.3390/bios12060413

**Published:** 2022-06-14

**Authors:** Benita Johannsen, Desirée Baumgartner, Lena Karkossa, Nils Paust, Michal Karpíšek, Nagihan Bostanci, Roland Zengerle, Konstantinos Mitsakakis

**Affiliations:** 1 Hahn-Schickard, Georges-Koehler-Allee 103, 79110 Freiburg, Germany; lena.karkossa@gmx.de (L.K.); nils.paust@hahn-schickard.de (N.P.); roland.zengerle@hahn-schickard.de (R.Z.); 2 Laboratory for MEMS Applications, IMTEK—Department of Microsystems Engineering, University of Freiburg, Georges-Koehler-Allee 103, 79110 Freiburg, Germany; desiree.baumgartner@hahn-schickard.de; 3 BioVendor-Laboratorní Medicína a.s., Research & Diagnostic Products Division, Karasek 1767/1, Reckovice, 62100 Brno, Czech Republic; karpisek@biovendor.com; 4 Faculty of Pharmacy, Masaryk University, Palackeho trida 1946/1, 61242 Brno, Czech Republic; 5 Section of Oral Health and Periodontology, Division of Oral Diseases, Department of Dental Medicine, Karolinska Institutet, 14104 Huddinge, Sweden; nagihan.bostanci@ki.se

**Keywords:** immunoassay, bound-free phase, micro/nanoparticles, point-of-care, centrifugal microfluidics, inflammation, reagent storage

## Abstract

In this paper, we present the ImmunoDisk, a fully automated sample-to-answer centrifugal microfluidic cartridge, integrating a heterogeneous, wash-free, magnetic- and fluorescent bead-based immunoassay (bound-free phase detection immunoassay/BFPD-IA). The BFPD-IA allows the implementation of a simple fluidic structure, where the assay incubation, bead separation and detection are performed in the same chamber. The system was characterized using a C-reactive protein (CRP) competitive immunoassay. A parametric investigation on air drying of protein-coupled beads for pre-storage at room temperature is presented. The key parameters were buffer composition, drying temperature and duration. A protocol for drying two different types of protein-coupled beads with the same temperature and duration using different drying buffers is presented. The sample-to-answer workflow was demonstrated measuring CRP in 5 µL of human serum, without prior dilution, utilizing only one incubation step, in 20 min turnaround time, in the clinically relevant concentration range of 15–115 mg/L. A reproducibility assessment over three disk batches revealed an average signal coefficient of variation (CV) of 5.8 ± 1.3%. A CRP certified reference material was used for method verification with a concentration CV of 8.6%. Our results encourage future testing of the CRP-ImmunoDisk in clinical studies and its point-of-care implementation in many diagnostic applications.

## 1. Introduction

Immunoassays can specifically detect protein biomarkers in various sample matrices such as serum, urine or saliva via the binding of an antigen by antibodies [1,2,3]. They are utilized in many diagnostic applications, ranging from infectious diseases such as malaria, dengue, respiratory tract infections or sepsis to non-communicable diseases such as cardiovascular and autoimmune diseases, periodontal disease, systemic inflammation and many more [1,2,4,5,6,7,8,9]. Most of these applications benefit from a shift from centralized laboratories towards the point of care (PoC) [2,5,10,11]. Such an approach significantly shortens the time from sample collection to test result, which can be crucial for many of the aforementioned applications, especially when acute and time-critical conditions are involved [12]. The implementation of tests at the PoC determines the following criteria from the end user’s perspective, and consequently defines technical requirements: (i) full automation of the analytical workflow so that the test can be performed even by untrained personnel, which requires that the test should have all the necessary (bio)chemical reagents pre-stored and should operate in a sample-to-answer manner; (ii) a rapid turnaround time (TAT), which necessitates a simple and short assay workflow with a low number and complexity of steps; (iii) capability for testing several samples per run (throughput), which requires that the assay reagents occupy as little space as possible on the fully integrated test cartridge; and (iv) optimally, the simultaneous detection (multiplexing) of different biomarkers, which requires the detection method to be compatible with multiplexing configurations.

Centrifugal microfluidic systems are suitable candidates for the shifting of diagnostic practice from centralized to PoC settings. Their closed cartridges can support fully automated analyses, do not require external pumps for fluid handling because they utilize changes in frequency and temperature to run their protocols, and are less prone to bubble clogging [13,14]. Pre-analytic protocols such as blood–plasma separation [15] and pre-treatment of whole saliva [16] can be easily implemented. These are some reasons why many different centrifugal system-based immunoassay solutions have been shown, using different assay methods and with different applications [17,18,19,20,21,22,23,24,25,26,27,28,29,30,31,32,33,34,35,36,37,38,39,40].

One main structural feature of most of the demonstrated immunoassays based on centrifugal systems is that they use magnetic or fluorescent micro/nanoparticles (in fact, only a few systems do not [18,22,26,28,39]). This is not surprising, as particles have been proven advantageous not only in miniaturized systems. Using magnetic particles as a solid phase shortens the incubation duration [41] and simplifies the handling during different assay steps, for example during washing [42]. The use of fluorescent beads as detection agents simplifies the realization of multiplexing, as a broad range of colors are commercially available [29,30], and they do not require any additional buffers, e.g., to activate and stop in case of an enzymatic reaction, while still retaining the required sensitivity [41,43,44].

The inclusion of micro/nanoparticles coated with proteins (either antibodies or antigens) as assay components requires solutions for their pre-storage directly on the cartridge in order to achieve fully automated sample-to-answer analysis at the PoC [13]. The pre-storage of proteins at room temperature (RT) is in itself a complex topic [45]. The pre-storage of proteins on beads and at RT further increases this complexity, because issues such as unwanted agglutination, resuspension efficiency and stabilization of the beads during the storage procedure are added to the equation. Additionally, as the protein-coupled beads must be stored inside the microfluidic cartridge, the chosen pre-storage process should be compatible with existing, scalable microfluidic cartridge manufacturing technologies. Despite the above challenges, this topic is barely addressed in existing literature. In fact, only a few publications on bead-based centrifugal microfluidic immunoassays report the pre-storage of reagents on the cartridge at RT [24,25,35]. However, none of these contain details on the topic or on the rationale behind the selection of the specific materials and protocols used, which makes it difficult to transfer and adapt their solution to different applications and different proteins. Among these, Lin et al. [24] and Lutz et al. [25] do not implement bead-based ELISA, but lateral flow-based assays with spotted detection lines, which include additional surface treatment steps or the insertion of a membrane into the cartridge, and therefore, their assays are, in their principle, fundamentally different to ours. Zhao et al. [35] automated a standard bead-based ELISA workflow, which, however, requires the pre-storage of four liquid components, includes absorption as a detection method (which does not allow multiplexing, as in the case of fluorescence), and detects ex situ after transferring the resulting liquid to a benchtop equipment. Moreover, all three approaches had to use some method to remove unbound reagents after the incubation in order to reduce the non-specific interactions during incubation and prevent a crosstalk of signal generation during detection. When using washing buffers, this has an impact on reagent consumption and the complexity of the microfluidic design, and although a few wash-free, bead-based configurations have been reported (Schaff et al. [30] and Gao et al. [40]), they implement an additional density medium on a centrifugal cartridge in order to achieve the wash-free operation.

In the context of the state of the art, we present a fully integrated centrifugal microfluidic system, the ImmunoDisk, implementing, for the first time, an immunoassay method that combines a bead-based and wash-free configuration without additional surface treatments or density media, with all reagents pre-stored, and using bound-free phase detection (BFPD). The bound-free phase detection immunoassay (BFPD-IA) is a heterogeneous assay based on ELISA principles and utilizes magnetic and fluorescent beads, the former acting as capture phase and the latter as the detection agent (further information on the BFPD-IA in Johannsen et al. [46]). The simplicity of the BFPD-IA concept allows the realization of assay incubation, bead separation and detection all in a single chamber. This reduces the overall footprint on disk, simplifies the liquid reagent pre-storage and makes the fluidic workflow more robust. Furthermore, the overall workflow of the BFPD-IA on disk and the fluidic structure are significantly simplified compared to the automation of standard ELISA workflow which, next to washing steps, needs to implement enzymatic reactions with the respective additional reagents [23,31,34,35]. Furthermore, in this work, detailed results of a parametric investigation on the pre-storage at RT of antibody-coated magnetic and antigen-coated fluorescent beads are shown for the first time, to the best of our knowledge, with the goal to use one common set of air drying conditions for a simplified disk production. For this, we investigated diverse drying buffer compositions by testing different additives and evaluated drying parameters such as temperature and duration.

The sample-to-answer ImmunoDisk performance in terms of robustness and reproducibility was assessed by detecting C-reactive protein (CRP) in human serum and by using a certified reference material (CRM). CRP was selected for demonstration due to its broad use as an inflammation biomarker [47] to support antibiotic stewardship. The properties of the competitive BFPD-IA on disk allow the direct usage of human serum without any pre-dilution steps in the clinically relevant range of 20 mg/L to 100 mg/L, as proposed by official guidelines in The Netherlands [48] or in the U.K. [49] for supporting clinicians on the decision of antibiotic prescriptions for patients at risk of pneumonia. This makes the ImmunoDisk attractive for the usage at the PoC for the detection of CRP, which is a highly concentrated marker in human serum [4,50,51].

Overall, our parametric study on the pre-storage of protein-coupled beads, in combination with the simplified, wash-free microfluidic integration of the BFPD-IA workflow, can open up new perspectives on the integration of heterogeneous, bead-based immunoassays within (centrifugal) microfluidic systems for user-friendly, rapid, sample-to-answer immunoassay-based diagnostics.

## 2. Materials and Methods

### 2.1. Bound-Free Phase Detection Immunoassay

The novel BFPD immunoassay was presented and evaluated in detail in a previous publication by Johannsen et al. [46]. The following is a brief summary of the procedure for the detection of CRP in human serum. The components of the competitive assay are the antibody-coupled magnetic beads as capture agents, the fluorescent beads coupled with the native competitive antigen as detection agents and one assay buffer (without any additional wash buffer). All reagents, together with the human serum sample or standards (spiked antigen concentrations in CRP-free human serum, HyTest, Turku, Finland), are added in a single reaction well (one-step assay) and incubated for 15 min at 37 °C. After this incubation, the signal from the unbound fluorescent beads, correlating to the concentration of CRP in the sample or standards, is measured.

In order to assess the activity of the dried bead-coupled antibodies and antigens after pre-storage and resuspension, we used a negative control of the CRP assay in a microtiter plate, as described in a previous publication [46]. This negative control assay includes both types of coupled beads without the native CRP antigen in the sample. Due to the nature of the BFPD-IA, the negative control facilitates an actual immunoassay reaction which, in the absence of native target analyte, is expected to lead to the maximum binding of the fluorescent bead-coupled competitive CRP antigen to the antibodies on the magnetic beads. Because the detection occurs in the (remaining) bound-free phase of the fluorescent bead suspension, such a negative control BFPD-IA gives the lowest signal, since no competition with any native antigen in the sample takes place.

### 2.2. Preparation of Magnetic Beads for the CRP Assay

The coupling of anti-human CRP antibodies (A80-125A, Fortis Life Sciences (Bethyl), Waltham, MA, USA) onto the tosyl-activated surface of the magnetic beads was described in detail in a previous publication by Johannsen et al. [46]. In short, the anti-human CRP antibodies were coupled onto the tosyl-activated surfaces of magnetic beads with a diameter of 2.8 µm (Dynabeads, M-280, Thermo Fisher Scientific, Waltham, MA, USA) overnight (18 h) at 37 °C under rotation. This was followed by a blocking step with 0.5% bovine serum albumin (BSA) (Carl Roth, Karlsruhe, Germany) for 2 h and a deactivation step with 50 mM ethanolamine (Carl Roth, Karlsruhe, Germany) for 1 h. After being washed twice, the beads were stored at 2.0% solid in a storage buffer (PBS (Thermo Fisher Scientific, Waltham, MA, USA), 0.1% BSA, 0.03% Synperonic P84 (Sigma-Aldrich, St. Louis, MO, USA), 0.05% sodium azide (Carl Roth, Karlsruhe, Germany)) at 4 °C until further use.

### 2.3. Preparation of Fluorescent Beads for the CRP Assay

The coupling of native CRP protein (C7907-26, 95–98%, highly purified, United State Biological, Salem, USA) onto the carboxyl-activated surfaces of the fluorescent beads (F8810, red (excitation 580 nm/emission 605 nm), 0.2 µm, Thermo Fisher Scientific, Waltham, MA, USA) was described in detail in a previous publication by Johannsen et al. [46]. In short, the carboxylated surface was activated with 25 mM EDC (1-ethyl-3-(3-dimethylaminopropyl)carbodiimide hydrochloride) (Thermo Fisher Scientific, Waltham, MA, USA) and 25 mM NHS (N-hydroxysuccinimide) (Thermo Fisher Scientific, Waltham, MA, USA). After activation, the CRP protein was coupled onto the surface in the presence of a 25 mM MES (2-(N-morpholino)ethanesulfonic acid) buffer with a pH of 6.1. A post-saturation step with 1.0% BSA and hydrolyzation of the remaining active groups with ethanolamine followed. After two washing steps, the beads were stored at 2.0% solid at 4 °C.

### 2.4. Parametric Investigation of Pre-Storage of Protein-Coupled Beads

For the evaluation of the drying buffer as part of the parametric study, 5 µL of drying buffer containing either the magnetic or the fluorescent beads was dried in a microtiter plate (96 wells, polystyrene, non-binding, Greiner Bio-One, Frickenhausen, Austria) in an incubator (INCU-Line IL 23, VWR International, Radnor, PA, USA) at 37 °C for 17 h and 6 h for the magnetic and fluorescent beads, respectively. They were stored in the dark in a container with silica beads. The beads were resuspended for 5 min at 750 rpm (BioShake iQ, QInstruments, Jena, Germany) before the CRP assay was conducted (see Section 2.1). The drying buffer was composed of different combinations of single or multiple additives.

### 2.5. Drying Buffer for the Magnetic Beads

The following additives to PBS were used (in different combinations) to test different drying buffer compositions for drying the anti-CRP antibody-coupled magnetic beads: (1) trehalose (Sigma-Aldrich, St. Louis, MO, USA), (2) sucrose (Sigma-Aldrich, St. Louis, MO, USA), (3) BSA, (4) Polyethylene glycol 1000 (PEG1000) (Merck KGaA, Darmstadt, Germany), (5) Tween80 (Sigma-Aldrich, St. Louis, MO, USA) and (6) CHAPS (Sigma-Aldrich, St. Louis, MO, USA). Additionally, two buffers from the companies Merck KGaA (Darmstadt, Germany) and GSK (Brentford, UK), which they use for the lyophilization of their proteins, were tested. The detailed composition of the additives in these buffers is given by Mensink et al. [45] (we dissolved them in PBS for comparison reasons). Two additional components that were not part of the overall parametric investigation, L-histidine (Sigma-Aldrich, St. Louis, MO, USA) and sodium phosphate dibasic heptahydrate (Sigma-Aldrich, St. Louis, MO, USA), were utilized for these buffers.

### 2.6. Drying Buffer for the Fluorescent Beads

The following additives to PBS were used (in different combinations) to test different drying buffer compositions for drying the CRP antigen-coupled fluorescent beads: sucrose, trehalose, PEG1000 and Tween80.

### 2.7. ImmunoDisk Cartridge Fabrication

The centrifugal microfluidic disks were designed using SOLIDWORKS 19 (Dassault Systèmes, Vélizy-Villacoublay, France) and simulated utilizing a network simulation processed with MATLAB Simulink Simscape R2016a (The Mathworks, Natick, MA, USA) that was improved for the development of centrifugal microfluidic disks, as described in detail by Schwarz et al. [52]. The disks were fabricated by means of thermoforming thin polycarbonate (PC) foils (Makrofol, thickness: 250 µm, Covestro AG, Leverkusen, Germany), as described by Focke et al. [53]. Instead of an elastomeric mold, as used by Focke et al., for the production of the cartridges in our work, a metal master tool was milled (EVO, KERN Microtechnik GmbH, Eschenlohe, Germany) and used for automatic production (Rohrer AG, Möhlin, Switzerland). The PC foil disks were punched to create an inner hole (10 mm diameter) and outer rim (130 mm diameter) that allowed exact placement of the disk on the processing device, the LabDisk Player 1 (DIALUNOX GmbH, Stockach, Germany). Magnetic and fluorescent beads were pre-stored on the cartridge in one step. The magnetic particles with anti-human CRP antibodies on their surfaces were pipetted from their stock, and the buffer was exchanged with the drying buffer (PBS, 10% (*w*/*v*) trehalose and 50% (*w*/*v*) PEG1000) to give a final bead concentration of 20 µg/µL. The fluorescent beads with native CRP antigen on their surfaces were diluted directly in their drying buffer containing PBS with 10% (*w*/*v*) trehalose and 10% (*w*/*v*) sucrose to give a final bead concentration of 3.8 µg/µL. Then, 5 µL of each of the magnetic and the fluorescent bead solutions was pipetted into dedicated storage chambers on the disk and then dried in an incubator at 39 °C for 1 h. The disks were stored at RT in a container with silica beads (SGT002, Silica Gel Shop, Haaksbergen, The Netherlands) before sealing. The assay buffer (dilution buffer, BioVendor, Brno, Czech Republic) was stored in stickpacks (described in detail by van Oordt et al. [54]) that are designed to open at a specific frequency on the disk. The disk was sealed with a pressure-sensitive adhesive film (9795R, 3M, Saint Paul, MN, USA). The vents and inlets were opened with a scalpel. The disks with all reagents pre-stored were kept in a container with silica beads at RT and protected from light.

## 3. Results and Discussion

### 3.1. ImmunoDisk—Description of Fluidic Workflow

**Figure 1:**
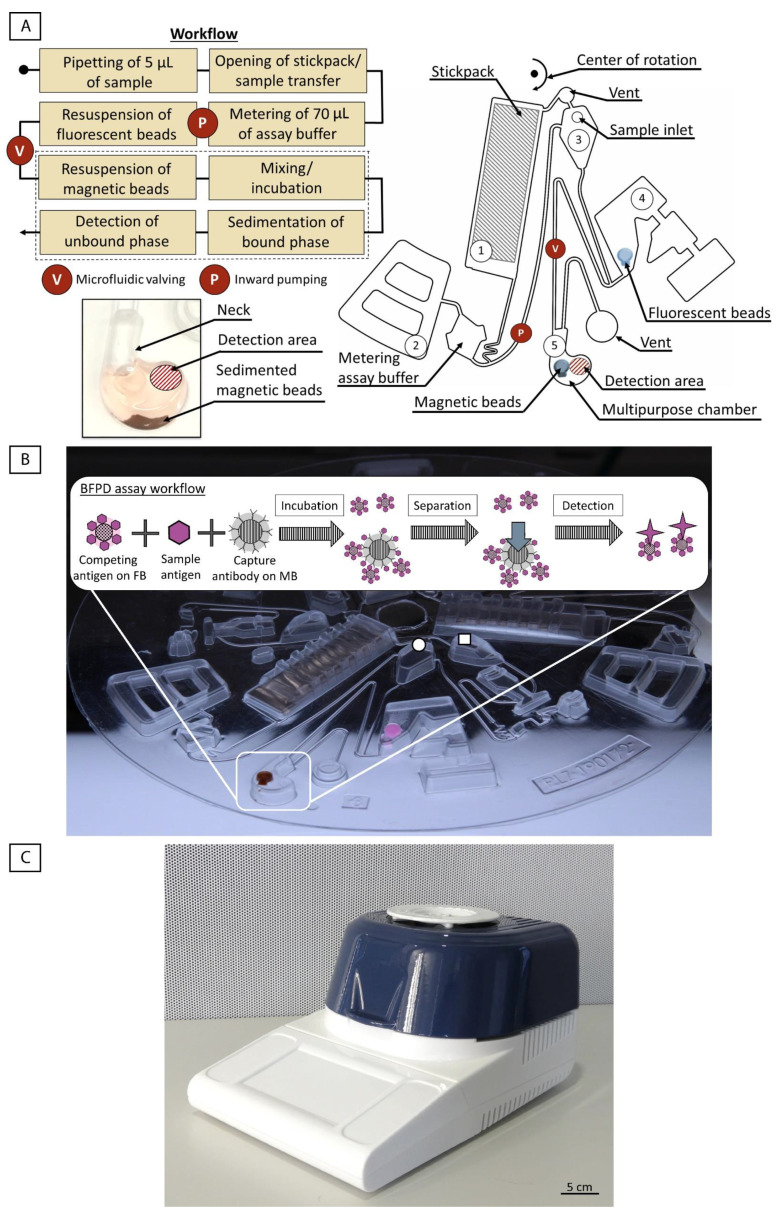
(**A**) Overview of the fluidic structure and workflow of the ImmunoDisk for the automation of the BFPD-IA. #1: stickpack chamber; #2: pneumatic valve chamber; #3: inlet chamber; #4: valve chamber; #5: multipurpose chamber. The workflow consists of only one inward pumping and one valving step. A specially designed multipurpose chamber (bottom left, #5) is used for storage of the magnetic beads, incubation (mixing), separation (sedimentation) and detection. The detailed fluidic workflow and the function of chambers #1–5 are described in Section 3.1. The parts of the workflow that are surrounded by a dashed line, all take place in the multipurpose chamber. (**B**) ImmunoDisk with stored magnetic (brown) and fluorescent beads (pink), shown here in liquid form before air drying, for better visibility. The assay buffer is stored in one stickpack that releases the buffer upon centrifugation. The schematic inset shows how the whole BFPD-IA workflow, including incubation, separation of the magnetic beads (MB) and detection of the unbound fluorescent beads (FB), takes place in the multipurpose chamber. Three complete structures fit on one cartridge. The drawn circle indicates the inlet for serum that was used in this study. The drawn square indicates the inlet for a potential whole blood sample, followed by a microfluidic module for plasma separation. This was not used in this study but was included in the design in order to assess the maximum amount of space consumed overall and for future usage. (**C**) Image of the processing PoC device (functional model), the LabDisk Player 1. The device was developed as a PCR cycler and can achieve different temperatures using air heating and is used to run centrifugal microfluidic disks. The device also contains two fluorescence detectors with four different colors in total.

A centrifugal microfluidic disk, the ImmunoDisk, was developed for the automation of the BFPD-IA workflow with all reagents pre-stored (Figure 1A,B). The assay buffer (110 µL) is stored in stickpacks, as previously described in detail [54]. The magnetic and fluorescent beads are stored in dedicated storage chambers via air drying. Details on the pre-storage process can be found in Section 3.5. An overview of the fluidic structure and the workflow is shown in Figure 1A. Three such fluidic structures can fit in a full disk, which allows the testing of three samples in parallel.

The disk is mounted into the processing device (LabDisk Player 1, see Figure 1C). Then, 5 µL of the sample (human serum) is pipetted into the inlet and the fluidic and temperature protocol is started (Supplementary File S1). The assay buffer in chamber #1 is released by the opening of the seal of the stickpack at a high frequency (80 Hz). Simultaneously, the sample flows from the inlet to chamber #4 (without yet resuspending the pre-stored fluorescent beads as the filling level is not high enough). The assay buffer then flows into a metering chamber to measure 70 µL out of the total 110 µL. Residual liquid is transported into the overflow chamber #2 and additionally loads the pneumatic pumping structure [55], which is activated by reducing the frequency to 7 Hz. The functionality of pneumatic valves and other pneumatic unit operations in centrifugal microfluidics is described in detail by Hess et al. [56]. The 70 µL of buffer is then pneumatically pumped radially inwards into the inlet chamber #3. With an increase in the frequency (40 Hz), the buffer is transferred to chamber #4, containing the stored fluorescent beads and the sample, which was transferred previously. This chamber is not vented, and the presence of liquid loads the valve. The fluorescent beads are resuspended in seconds upon mixing with the sample and the assay buffer. The valve is activated again by reducing the frequency (14 Hz) and by increasing the temperature to 37 °C, which is also the incubation temperature for the immunoassay. The liquid is then transferred into the multipurpose chamber #5, resuspending the magnetic beads. Batch-mode [57] mixing during incubation is started. No external magnets are required to achieve a sufficient mixing result. After incubation, the frequency is increased (30 Hz for 30 s, 40 Hz for 10 s) to sediment the bound-phase and separate it from the bound-free phase (Figure 1A and a photo of the sedimented bound-phase can be found in Supplementary File S2). The disk is stopped so that the multipurpose chamber is located above the detector for the fluorescence signal readout in the bound-free phase. The detection area is well-defined and spatially separated from the sedimented bound phase to prevent any interference with the signal.

### 3.2. Fluidic Characterization

Some variance in the entire fluidic system, and consequently the measurement result, may in principle derive from volume variances when (i) pipetting the sample into the inlet or pipetting the liquid solutions of fluorescent and magnetic beads prior to drying, (ii) metering the assay buffer, (iii) pumping the assay buffer radially inwards into chamber #3 and (iv) valving and transferring the mixture (sample, assay buffer and resuspended fluorescent beads) from chamber #4 into the multipurpose chamber #5.

The performance of the metering process and the volume transfer of the pneumatic pumping and the pneumatic valving steps were evaluated by pipetting 5 µL of assay buffer into the sample inlet and 110 µL of assay buffer into the stickpack chamber. The fluidic protocol was started and the volume that was transferred into the incubation chamber was measured (N = 6) with an average of 78.3 ± 0.5 µL. This corresponds to a deviation of 4% from the targeted 75 µL and shows a high reproducibility, with a coefficient of variation (CV) below 1%. To assess the reproducibility of the resuspension of the fluorescent beads and the fluidic valving, fluorescent beads were dried in their assigned storage chamber #4 before 75 µL of buffer was pipetted into the inlet to ensure a controlled volume. The buffer was transferred to chamber #4 containing the fluorescent beads, which were resuspended, mixed and transferred to the multipurpose chamber. After batch-mode mixing for 200 s, the fluorescence signal was measured (N = 9). The average signal of 1132 ± 30 RFU showed a CV of only 2.7%. Part of these (anyway small) variances may be attributed to the manual pipetting and variances of the detector. We expect even lower variances for automatically produced disks in the future. The combination of pneumatic and temperature-induced overpressure during valving ensures that all of the resuspended fluorescent beads are transferred to the multipurpose chamber. All these results demonstrate low variation deriving from the microfluidic operations and show that the disk and its fluidic protocol are well-suited for performing the BFPD-IA.

### 3.3. Multipurpose Chamber

**Figure 2:**
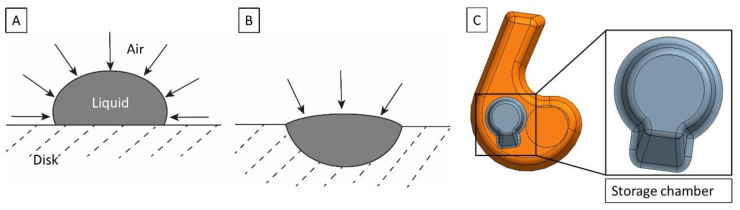
(**A**) Droplet for drying, positioned on the disk planar surface without a storage chamber. The air–liquid interface is defined by the properties of the drying buffer and its contact angle on the cartridge surface. (**B**) Droplet for drying in a dedicated storage chamber on the disk. The size of the air–liquid interface is reduced. (**C**) Multipurpose chamber (orange) with storage chamber (gray) and detection area (black circle). The storage chamber has a volume of 5 µL and includes a ramp that supports the flow of beads radially outwards after resuspension.

The multipurpose chamber was designed with some special features to facilitate the bead and liquid handling and to optimize the various assay steps, all in a single chamber (Figure 1A). (i) A radially inward ‘neck’ was introduced to the chamber in order to stabilize the meniscus that forms when the disk stops below the detector for the readout step. The assay buffer used for the detection of CRP in human serum has a contact angle of 74° (measured with Physica MCR101, Anton Paar, Graz, Austria) on the polycarbonate disk material, which makes it wetting (as its contact angle is <90° [58]). (ii) The multipurpose chamber incorporates a small second chamber, which is used for the storage of the magnetic beads (storage chamber, Figure 2C). Notably, the chamber has been positioned a distance away from the planar detection area in order to not interfere with it (Figure 1A). (iii) The radially outward rounding facilitates the collection of the sedimented magnetic beads (Supplementary File S2) and prevents them from interfering with the detection, which is realized within the same chamber.

A key element of the BFPD-IA method is the detection in the unbound phase, which consists of only the fluorescent beads, after the bound phase (the complexes of magnetic beads with fluorescent beads and/or CRP antigen from the sample) has been removed. Due to its special features, and in combination with the appropriate centrifugal protocols, the multipurpose chamber allows this key element of the BFPD-IA to be transferred to the disk cartridge by integrating the single-step assay incubation (including mixing), the separation of the bound phase (sedimentation) and the detection in the same chamber. This is enabled by the fact that, based on the design of the assay itself, the unbound fluorescent beads are not sedimented, while the bound phase is. The distance traveled by a bead at specific frequencies can be estimated using Stokes’ law modified for centrifugal forces [59,60] (equation in Supplementary File S3, where it is shown that the bead radius and density are the key parameters). The extreme case scenario for the sedimentation calculation would consist of a single magnetic bead (nothing bound on its surface) at the most radially inwards position in the multipurpose chamber, and thus traveling the maximal distance of 0.5 cm to a radial position of 6.2 cm. The fluidic protocol consists of two sedimentation steps. The first step sediments with 30 Hz for 30 s and the second step with 40 Hz for 10 s. Using the equation in Supplementary File S3 to calculate the distance traveled gave the final position of a single magnetic bead at 6.2 cm. We applied both sedimentation steps to a single, unbound fluorescent bead, and we calculated that it would move only a few micrometers. Thus, we can be sure that the fluorescent beads are not subjected to sedimentation when using our protocol. Overall, the capability to perform our assay and readout in a single multipurpose chamber has significant positive consequences, as it (i) saves time, (ii) reduces possible errors (no bead loss, metering is omitted, no liquid transfer) and (iii) reduces the occupied space, enabling more structures to fit on one disk. This can subsequently increase the throughput and reduce the manufacturing costs.

### 3.4. Storage Chamber on the Disk

There can be significant denaturation of proteins at the air–liquid interface during the drying for pre-storage [61]. The air–liquid interface can be reduced by placing the bead-containing solution into a geometrically confined area, rather than simply on the planar surface of the plastic cartridge (Figure 2), thereby reducing the risk of protein denaturation. We have also observed that a droplet on the cartridge surface can become unstable during handling. This leads to the liquid spreading over the surface and a considerable increase in the air–liquid interface. Therefore, the ImmunoDisk consists of two storage chambers (close-up of the design in Figure 2C) that are used to pre-store the fluorescent and magnetic beads directly on the cartridge. Such storage chambers simplify the pipetting and handling during production and allow a more robust fabrication of the complete cartridge (also advantageous for transport) because the air–liquid interface is more stable in the storage chamber than on the planar surface. Furthermore, before we started using the storage chamber, when we simply pipetted the bead solution onto the planar cartridge surface, pellets occasionally broke or became loose during handling after drying. This did not occur after we started using the storage chamber. We also observed better resuspension behavior of the magnetic bead pellet when the storage chamber was used, because of a ramp that supports the flow of beads radially outwards during resuspension (Figure 2C).

### 3.5. Study on Pre-Storage of Protein-Coupled Beads

Reagent pre-storage on a disposable microfluidic cartridge is essential in order to provide full automation. It reduces the number of hands-on steps and allows easy transportation of the disposable cartridges. Storage of proteins, especially at RT, is challenging. Factors such as heat, shear forces and contact with interfaces can result in a loss of protein functionality due to denaturation [45,61]. There are some theories on how proteins can be protected against denaturation during storage, which can be summarized in two categories: either the mobility of a protein (vitrification theory) or reactions with its surroundings (water replacement theory) must be restricted [45]. Lyophilization or freeze-drying is often used to store proteins (alone, i.e., without beads) due to the good stabilization achieved with this method, even though it is costly and technologically complex [61]. Concerning beads alone, we have demonstrated protocols for pre-storage in the case of magnetic beads for nucleic acid extraction and purification [62,63,64]. However, the BFPD-IA that is integrated within the ImmunoDisk involves protein-coupled magnetic and fluorescent beads. This results in specific requirements and also restrictions for the selection of the pre-storage method. As the fluorescent beads that were used in this work cannot withstand freezing, according to the manufacturer [65], the lyophilization approach was not an option. Storing the protein-coupled beads in liquid buffer was also excluded, in order to avoid cold chain requirements during transport. Therefore, the air drying method was chosen.

From a scalable manufacturing perspective, it was also judged that air drying would be a simpler workflow compared to lyophilization (i.e., the creation of a pick-and-placeable lyopellet), because the former can be conducted with dispensing robotics and large ovens, while the latter would require a pick-and-place robot and strict environmental humidity conditions.

The requirements for the pre-storage on the ImmunoDisk (and also for bead-based PoC immunoassays in general) were (i) maintaining the functionality of the proteins, (ii) the fast and complete resuspension of the beads and (iii) the prevention of aggregate formation. Additional manufacturing-related requirements demand that there should be (iv) stable bead pellets and, ideally, (v) no additional treatment of the (cartridge) surfaces.

The parameters that may influence the storage result are the drying buffer (including protein stabilizing additives), the drying temperature and the drying duration next to the fixed parameters of bead type, proteins and volumes. Thus, we conducted a detailed experimental investigation of these parameters. In the first step, we tested a non-exhaustive list of candidate drying buffers, temperatures and durations and evaluated each of them by means of assay performance and pellet resuspension. We also took into account that the final drying conditions should be compatible with both bead types (magnetic and fluorescent), as well as both biomolecule types coupled on the beads’ surface (anti-human CRP antibodies and native CRP antigens, respectively).

This part of our work should serve as an orientation for the reader in the field of pre-storage of protein-coupled beads. We make no claim of a complete investigation of the drying parameters and conditions, nor was the goal to examine the impact of these conditions on the structure/function of the proteins and beads.

### 3.6. Parametric Study on Drying Buffers

We started our study by investigating suitable drying buffers first for the antibody-coupled magnetic beads (MB), while keeping the antigen-coated fluorescent beads (FB) in solution, and then for the antigen-coupled fluorescent beads, while keeping the antibody-coated magnetic beads in solution. We used the BFPD-IA, where all reagents were in the liquid state, as a reference. The signal intensity resulting from each assay performed with a specific drying buffer was subtracted from the reference and this ΔIntensity was used to assess the functionality of the reagents after drying (as ΔIntensity shows the difference from the reference, it should be as low as possible). A higher CV could indicate the formation of aggregates or unreproducible resuspension properties and therefore, the CV should be comparable to the CV of the reference assays. Images of dried beads were taken after drying with the attempt to find a correlation between the visual properties of the pellet and the post-rehydration assay performance, but we did not find any. Therefore, we considered only the assay performance itself as an evaluation criterion for the drying process. These evaluation experiments were first performed on a microtiter plate, and the best-performing drying buffers were then tested on the cartridge.

Representative additives were selected from classes of reagents that have been reported to support the stabilization of proteins, such as sugars, polymers and detergents [45,61], or to counteract possible adsorption effects, such as BSA [66].

**Figure 3:**
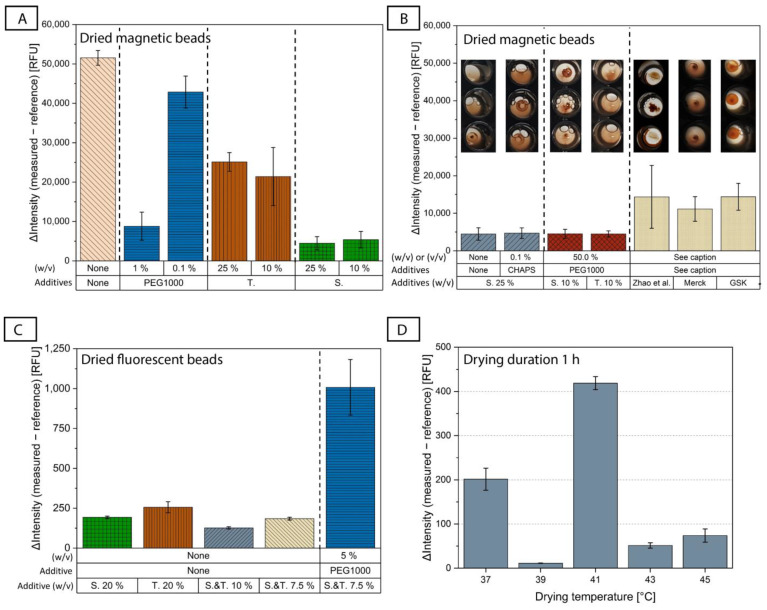
(**A**) Comparison of the assay behavior after air drying of magnetic beads (keeping fluorescent beads in solution) with some representative drying buffers based on PBS and containing only one additive. The additives tested were trehalose (T.), sucrose (S.) and PEG1000, as well as only PBS (‘none’). ΔIntensity evaluates the assay performance. N = 3, except S. 25%, which already includes all measurements conducted during the course of this study, for which N = 10. (**B**) The four best-performing drying buffers for magnetic beads from this parametric study were measured again on a second day using more repetitions to ensure reproducible results with a total of N = 10. To show different resuspension behavior, the best-performing buffers are compared to the buffer published by Zhao et al. [35] and buffers from the companies Merck and GSK that were developed for lyophilization of proteins (N = 3; their compositions are given in [45]). The figures above the bar diagrams show the differences in the resuspension of the dried magnetic beads (for more repetitions than N = 3, representative figures were chosen). (**C**) Comparison of the assay behavior after air drying of fluorescent beads (keeping the magnetic beads in solution) with some representative drying buffers (N = 3). (**D**) An overview of the assay performance with different drying temperatures (37 °C (N = 3), 39 °C (N = 4), 41 °C (N = 2), 43 °C (N = 5) and 45 °C (N = 2)) and a common drying duration of 1 h using the chosen drying buffers for magnetic beads (trehalose 10% (*w*/*v*), PEG1000 50% (*w*/*v*) in PBS) and fluorescent beads (trehalose and sucrose 10% (*w*/*v*) in PBS) on the disk. Colors of the bar diagrams are included for better visualization and are not intended for cross-correlation between the figures (**A**) to (**D**).

Different drying buffer combinations were tested for the air drying of MB, and the most representative results are shown in Figure 3A. We started by using PBS, which is used as the base for the drying buffers, alone without additives (listed as ‘none’). We observed an (undesirable) high ΔIntensity signal. This indicates that either the beads could not be resuspended completely or the proteins on the surface of the beads lost their binding affinity.

Two different disaccharides were tested, trehalose and sucrose. Disaccharides are non-reducing sugars and, in contrast to reducing sugars, do not denature proteins with a Maillard reaction [61]. It has also been shown that they can stabilize proteins during storage [45]. For the MB, the ΔIntensity was clearly lower when adding trehalose and sucrose at different concentrations than when using PBS alone (Figure 3A), indicating the positive effect that both disaccharides had on the preservation of the proteins’ functionality, with sucrose appearing to perform better. We did not observe any major difference in the ΔIntensity between 10% and 25% (*w*/*v*) for either of the two disaccharides.

We also examined a representative polymer additive, PEG1000. PEG as a protein stabilizer has been controversially discussed in the literature and is often said to be used with caution, as its effect is protein-dependent [61,67,68]. While some of its properties, such as the good hydration of proteins in PEG-containing liquids and its ability to adsorb to the hydrophobic core of proteins, indicate good stabilizing potential [69,70], it has also been shown that its usage reduces protein transition temperatures, above which the structure of a protein changes [68]. Experimentally, we saw that when using PEG1000 alone at a concentration of 1% (*w*/*v*), we had quite low ΔIntensity values, almost as low as in the case of sucrose (Figure 3A), which implies that it can stabilize the bead-coupled antibodies even at this low concentration. In contrast, lower PEG1000 concentrations of the order of 0.1% had a sub-optimal performance. However, we observed that 1% (*w*/*v*) PEG1000 combined with 10% (*w*/*v*) trehalose or 10% (*w*/*v*) sucrose was not sufficient to improve the results, which were ~3× worse when using the sugars alone (Supplementary File S4A). When we increased the PEG1000 concentration to 25% (*w*/*v*) and combined it with 10% (*w*/*v*) sucrose or 10% (*w*/*v*) trehalose, the ΔIntensity was drastically reduced, although with some large error bars for the case of 10% (*w*/*v*) sucrose/25% (*w*/*v*) PEG1000 (Supplementary File S4A), which was solved when further increasing the PEG1000 concentration to 50% (*w*/*v*) (Supplementary File S4A and Figure 3B). This could lead to the conclusion that if PEG1000 is used as a second additive alongside a disaccharide, its concentration needs to be high for it to have an impact. Another important observation is that although the sugars performed differently when used alone (e.g., 10% (*w*/*v*) sucrose vs. 10% (*w*/*v*) trehalose, Figure 3A), this difference was no longer observed when PEG1000 was added at a concentration of 50% (Figure 3B, red columns).

Three further additives were combined, in different concentrations, with both 25% (*w*/*v*) trehalose and 25% (*w*/*v*) sucrose: BSA, which might help to reduce non-specific binding to surfaces during drying [66], and the detergents Tween80 and CHAPS (in powder form at RT), which could prevent the adsorption and aggregation of proteins [61]. According to our results, the addition of BSA did not have a positive effect (Supplementary File S4B,C). We also observed different behavior between Tween80 and CHAPS (Supplementary File S4B,C), which shows that the type of detergent has an influence. Neither improved the results when added to sucrose, but adding 2% (*v*/*v*) Tween80 to trehalose reduced the ΔIntensity substantially, although the CV was increased.

Figure 3B shows a comparison of the four buffers which performed best for the drying of MB in this parametric study. We found that 25% (*w*/*v*) sucrose, 25% (*w*/*v*) sucrose with 0.1% (*w*/*v*) CHAPS, 10% (*w*/*v*) sucrose with 50% (*w*/*v*) PEG1000, and 10% (*w*/*v*) trehalose with 50% (*w*/*v*) PEG1000 had similar ΔIntensity results. As mentioned earlier, the selection of the drying buffer should be based not only on the degree of stabilization of protein function (assessed through the assay performance), but also on the quality of the resuspension of the dried magnetic bead pellet. The corresponding photo of the well after resuspension of the dried MB is shown in Figure 3B to emphasize the different resuspension behavior depending on the drying buffer used, even though all four buffers showed similar ΔIntensity results. This could indicate that the performance criteria of measured ΔIntensity and its CV represent the combination of the resuspension behavior of the pellet, the protein stabilization properties of the drying buffer and the formation of bead-aggregates after resuspension of the pellet.

We also tested commercial buffers from the companies Merck and GSK [45] that were developed for lyophilization of proteins, as well as the buffer by Zhao et al. [35]. However, these did not offer any significant improvement compared to the drying buffers from our study, neither in terms of assay performance (indicated by the high ΔIntensity), nor in terms of bead resuspension (last three inset photos in Figure 3B).

After the screening of different drying buffers in the microtiter plate, the best-performing buffers for the drying of protein-coupled magnetic beads were tested directly on the ImmunoDisk cartridge (exemplary results in Supplementary File S5). The final buffer that was chosen for use in further experiments was 10% (*w*/*v*) trehalose with 50% (*w*/*v*) PEG1000 in PBS. It showed superior resuspension quality and reproducibility in the cartridge, as was the case in the microtiter plate (Figure 3B).

The parametric study continued with the investigation of drying buffers for the antigen-coupled fluorescent beads, while keeping the magnetic beads in solution. The parametric study for the drying of native CRP-coated fluorescent beads included fewer candidate drying buffers than in the tests for the magnetic beads, as we utilized findings from the latter study. We did not observe as big a difference between trehalose and sucrose (Figure 3C). While PEG1000 showed promising results for the CRP-antibody coupled magnetic beads, it resulted in an increase in the ΔIntensity after drying of the antigen-coupled fluorescent beads (Figure 3C and Supplementary File S6). The CRP native antigen that is coupled onto the fluorescent bead surfaces is mainly hydrophobic [71], and it has been shown that hydrophobic proteins can be destabilized by PEG [68]. These findings demonstrate that there is no universal solution for additives in drying buffers for different types of proteins (antibody vs. CRP antigen), and that candidate buffers should be tested for each protein individually. The addition of Tween80 at various concentrations also did not improve the results (Supplementary File S6).

Due to the above, we decided to continue with the drying buffer for FB containing only sugars. Figure 3C shows that 10% (*w*/*v*) trehalose combined with 10% (*w*/*v*) sucrose in PBS gave the lowest ΔIntensity value, and therefore, this drying buffer was selected for the fluorescent beads.

The fact that different drying buffers had to be selected for the antibody-coated magnetic beads and the antigen-coated fluorescent beads might be due to the different nature of the proteins, which we also observed with the different impact of PEG1000 on the drying results. The drying buffer for the CRP antibody-coupled magnetic beads also showed promising results when used for magnetic beads that were coupled with matrix metalloproteinase 9 (MMP-9) antibodies [46] measured on disk (Supplementary File S7). The fact that the same magnetic bead-drying buffer was used successfully with two different antibodies could be a preliminary indicator that it can be used for a range of different antibodies. This should be investigated further in the future.

Our experimental observations indicate that for different types of proteins, it is necessary to test different additives in different concentrations, and that there is no universal solution for all proteins.

### 3.7. Investigation of Pre-Storage Temperature and Duration Conditions

Having defined the biochemistry of the drying buffers for the magnetic and fluorescent beads, the next step in our parametric study was to define the drying conditions. Unlike the buffers, which do not have to be the same, the duration and temperature must be the same for both drying buffers, as the two bead solutions must be dried simultaneously and in the same cartridge.

We used a design of experiments (DoE) approach with Minitab (Minitab GmbH, Munich, Germany) to evaluate the influence of temperature and duration on the drying of the protein-coupled beads. The DoE showed that the combination of drying temperature and duration has the strongest influence on the result, along with the drying duration alone. The drying temperature by itself has no significant influence on the result. It should be kept in mind that the DoE tested low and high temperatures of 37 °C and 45 °C and that higher temperatures can have a strong influence, for example, if the proteins are denatured. For more information on the DoE please see Supplementary File S8.

The drying duration was set to 1 h, which is favorable from a disk manufacturing perspective. A screening of different drying temperatures, shown in Figure 3D, was conducted to find the best-performing temperature–duration combination. The drying was carried out directly on the ImmunoDisk (in the storage chambers, Figure 1A) and not in the microtiter plate wells. We observed differences in the ΔIntensity and the signal CVs for different drying temperatures (Figure 3D). The negative control that performed best, showing a low CV (3.3%) and a low ΔIntensity (11.3 RFU), was dried at 39 °C. All other temperatures led to either higher CVs (3.4–20.3%) or a higher ΔIntensity. Thus, the final selected drying temperature and duration were 39 °C and 1 h, respectively. Some additional screening with longer drying durations at different temperatures was conducted, which ensured that the drying at 39 °C for 1 h indeed shows superior performance compared to longer drying durations (overview of the data in Supplementary File S9). Using the drying conditions of 39 °C for 1 h, the resuspension was completed in a few seconds for the fluorescent beads and in less than 90 s for the magnetic beads.

Having specified the drying buffers and conditions, we briefly explored the short-term stability (two weeks) of the dried beads in a disk cartridge at RT. We first used dried magnetic beads and liquid fluorescent beads. We compared the average results on day 1 and day 16 using the negative control. In this configuration, we observed that the signal on day 16 after drying differed by 1.7% compared to that on day 1. In a subsequent set of experiments, we used dried magnetic beads as well as dried fluorescent beads. As above, we used the negative control. The negative control was measured after 1, 4, 11 and 15 days of storage at room temperature. In this case, the average signal on day 15 after drying differed by 4.7% compared to day 1. The measurements over the four different days after storage show a variation of 6.3% (N = 7). These are promising preliminary results and the deviations are within the inter-assay variation. The storage stability over time needs to be evaluated further with longer storage durations and over all concentrations covered by the calibration curve, but this would go beyond the scope of this work.

### 3.8. Sample-to-Answer CRP Detection on the ImmunoDisk

In a final step, the results from the developments in the areas of reagent pre-storage and assay transfer to microfluidics were demonstrated in a sample-to-answer configuration on the disk with all reagents stored at RT. CRP was used as a target antigen and CRP-free human serum as a complex matrix.

**Figure 4:**
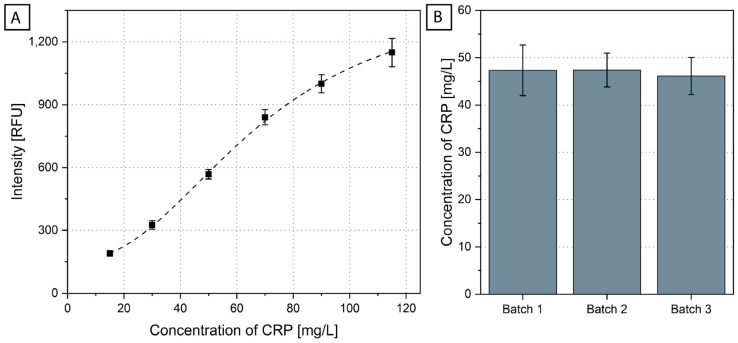
(**A**) A calibration curve (N = 6 for 50 mg/L, N = 7 for all other concentrations) for the system was obtained by running several concentrations on disks produced in three different batches. It is normalized to the overall blank (results of each batch are shown in Supplementary File S10). (**B**) Averages and standard deviations of the CRP concentration in the CRM sample were measured using three batches of disks (N = 11 measurements in total, batch 1 N = 4, batch 2 N = 3, batch 3 N = 4). The calculation of the CRP concentration was based on the calibration curve of (**A**) and the 4-parameter fit shown in Supplementary File S10. The 11 CRM measurements are shown in Supplementary File S11.

A calibration curve was obtained by measuring CRP-spiked human serum standards using three batches of disks on three different days (Figure 4A). This allowed us to perform a reproducibility assessment, which showed an average inter-assay signal CV of 5.8 ± 1.3%. The variation was below 10% and included the influence of the pre-storage of the protein-coupled beads at RT, fluidic variations and production variation. This shows a high reproducibility of the BFPD measurement for CRP in the range between 15 mg/L and 115 mg/L, which includes the clinically relevant cut-off values [51,72]. The limit of detection (LOD) was calculated as 18.2 mg/L. Although some central laboratories use 5 mg/L as a low cut-off value of CRP for general inflammation conditions, clinical studies focusing on specific health conditions and especially respiratory tract infections use ‘zones’ of CRP concentrations for supporting the decision of prescribing antibiotics or not. In particular, Prins et al. [51] and Jakobsen et al. [72] report a single cut-off value of 50 mg/L, and Alcoba et al. [73] report 80 mg/L. Eccles et al. [49] and Schuijt et al. [48] report two cut-off values, 20 mg/L and 100 mg/L. Thus, the range that the ImmunoDisk covers is compatible with these application-specific and clinically relevant cut-off values. Additionally, these concentration ranges and LOD values obtained using the fully integrated ImmunoDisk are in good agreement with the obtained range and LOD when the BFPD-IA was conducted on a benchtop laboratory instrument [46]. Furthermore, the TAT of the ImmunoDisk for the detection of CRP is 20 min and three samples can be processed in parallel.

The obtained calibration curve (information on the fit of the curve in Supplementary File S10) was used to calculate the concentration of CRP in a CRM (European Commission Joint Research Centre, Geel, Belgium; ERM-DA474/IFCC). Three different batches of disks were again produced and were used for the measurement of CRM (Figure 4B). The CRM was measured 12 times. One measurement led to an outlier (see Supplementary File S11 for the calculation of the outlier), which could stem from handling variations in one or more steps during the currently manual production of the disks, including the insertion of the stickpack, the pipetting of the beads or the sealing of the disk. These sources of errors will be reduced when the cartridge is scaled up to mass production (notably, all steps of the manufacturing process are compatible with an automated production line [74]). The expected value of the CRM was 41.2 ± 2.5 mg/L, measured using immunonephelometry and immunoturbidimetry, without pre-stored reagents [75]. The average measured value on the ImmunoDisk was 46.9 ± 4.0 mg/L (N = 11). A deviation between measurements with different systems and detection principles can be expected. For diagnostic applications where CRP is used as biomarker to support antibiotic stewardship, it is sufficient to provide the CRP concentration between/beyond cut-off values that are reported in clinical studies and guidelines [48,49,72,73]. In any case, the usability of the test for diagnostic purposes needs to be evaluated in a clinical study, which is outside the scope of this work. In the context of our technical feasibility assessment, the inter-disk concentration CV in our case was only 8.6% (calculated over all batches). Moreover, the three different ImmunoDisk batches showed great reproducibility, with an inter-batch CV of 1.5% calculated using the average measured concentration of each batch.

### 3.9. Overall Assessment of the ImmunoDisk

The overall goal was to fully integrate (including pre-storage of reagents) the BFPD-IA on a microfluidic cartridge. Notably, the initial version of the BFPD-IA, conducted in a microtiter plate, included a step to transfer the bound-free phase to a separate well for detection after the separation step. However, through the interplay between microfluidic design and protocols, we managed to not only reduce the manual handling steps to just one (insertion of the sample), but also to omit the transfer of the bound-free phase and to achieve incubation, separation and detection all in the multipurpose chamber. Thus, the few and easy steps of the BFPD-IA itself [46], together with smart microfluidic solutions, enabled a simple transfer of the assay to a space-saving microfluidic design. This also has a potential impact on the test throughput. Specifically, in our case, three identical structures (including the blood–plasma separation module that was not used in this work) could fit on a single disk, which means that in the future, three patients can be screened simultaneously. The simple structure of the disk, which utilizes a multipurpose chamber approach for multiple operations, means that no additional sensors, extra components or surface treatment are required for integration of the BFPD-IA method. This demonstrates how the simplicity of a biochemical assay protocol like the BFPD-IA, which has a single step, is wash-free and rapid, can be crucial for the sustainability, viability, robustness and cost of a PoC system.

For the full integration of a bead-based immunoassay PoC system like the one we propose, it is inevitable that the pre-storage of protein-coupled beads comes into focus. The parametric study of this topic presented in this publication revealed some interesting findings.

We show for the first time, to the best of our knowledge, the combination of a polymeric and a disaccharide as additives for the pre-storage of protein-coupled beads using air drying. We observed that the buffer with a combination of PEG1000 and trehalose performed best for the CRP antibody-coated magnetic beads, but that PEG itself proved to be incompatible with the CRP antigen-coated fluorescent beads, most probably due to the different nature of the coupled protein. The common drying protocol for the two different types of beads and proteins on the same disk is also an important outcome. This can open the way for other bead-based assays to be integrated in microfluidic systems.

In its fully integrated version, the ImmunoDisk is able to measure high concentrations of CRP in a 5 µL human serum sample, which is inserted directly into the cartridge with no prior dilution steps and no washing steps on disk. These conditions simplify the fluidic integration and reduce the footprint on disk. Importantly, the ImmunoDisk was processed on a device, the LabDisk Player 1 functional model, which was developed for the automation of nucleic acid amplification technologies (NAATs, e.g., PCR and isothermal methods) and for which different applications have already been shown (e.g., respiratory tract infections [64], tropical infections [63], oral diseases [62] and vector analysis in mosquitos [76]), without any changes to the device hardware. The compatibility of the ImmunoDisk with this NAAT device is primarily due to the protocol and the detection principle of the BFPD-IA, as well as the adaptation of the microfluidic structures. This may prospectively pave the way for the more general implementation of the BFPD-IA on other NAAT devices, thereby expanding their portfolio and achieving complementary diagnostics where, for example, pathogen identification and immunological response are tested on the same instrument, with major impacts on the health economics of diagnostics. Moreover, the health systems in several areas of medicine that require such co-assessment and interoperability between microbiological and immunological outcomes [50,73,77,78,79,80] would benefit from such complementary diagnostics.

## 4. Conclusions and Outlook

In this work, we show, for the first time, the full sample-to-answer integration and automation of a heterogeneous wash-free, bead-based, bound-free phase detection immunoassay for the detection of CRP in serum in the clinically relevant range, including in situ detection and pre-storage of all involved reagents. The results show the great potential of the ImmunoDisk to be processed on the same centrifugal microfluidic processing device that performs PCR or isothermal amplification.

For the pre-storage of the protein-coupled beads, we investigated all the key parameters for the air drying of proteins on particles and gained some key insights on the influence of additives and the importance of the drying temperature and duration combination. Thus, this work contributes significantly to the research methodology around the pre-storage of beads coupled with proteins, for which there is, to the best of our knowledge, limited literature available, unlike for proteins or beads alone. From the microfluidic perspective on the pre-storage of protein-coupled beads, we propose using a storage chamber on the cartridge for a more reproducible and robust storage and release of the protein-coupled beads. Furthermore, a multipurpose chamber is introduced that facilitates the assay incubation, separation and detection, leading to a simplified, robust, space-saving microfluidic design. The future outlook for this work includes the expansion of the pre-storage protocols to other protein assays, covering applications such as oral health, cardiovascular diseases or sepsis, and the implementation of the CRP-ImmunoDisk with clinical samples and potentially in combination with molecular diagnostics for improved patient management at the PoC.

## Data Availability

Data are contained within the article or in the Supplementary Material.

## Supplementary Materials

The following supporting information can be downloaded at: https://www.mdpi.com/article/10.3390/bios12060413/s1, File S1: ImmunoDisk—fluidic protocol; File S2: Sedimentation of magnetic beads; File S3: Calculation of the distance traveled by a bead at specific frequencies; File S4: Results of parametric study for the drying of magnetic beads; File S5: Magnetic bead resuspension experiments on disk; File S6: Overview of different drying buffers for native CRP-coupled fluorescent beads; File S7: MMP-9 assay with dried antibody-coated magnetic beads and liquid antigen-coated fluorescent beads; File S8: Design of Experiments (DoE); File S9: Overview of ΔIntensity and CV using different drying temperatures and durations; File S10: Calibration curve; File S11: Data of CRM measurements and outlier calculation.
