# Supplementary material for: ImmunoDisk—A Fully Automated Bead-Based Immunoassay Cartridge with All Reagents Pre-Stored
Source: Biosensors (Basel). 2022 Jun 14;12(6):413. doi: 10.3390/bios12060413 (PMC9221266; doi:10.3390/bios12060413)
Supplement: Supplementary file 1 [file biosensors-12-00413-s001.zip › biosensors-1747176-supplementary.pdf]

Supplementary Materials

ImmunoDisk—A Fully Automated Bead-Based Immunoassay Cartridge with All Reagents Pre-Stored

File S1. ImmunoDisk—fluidic protocol

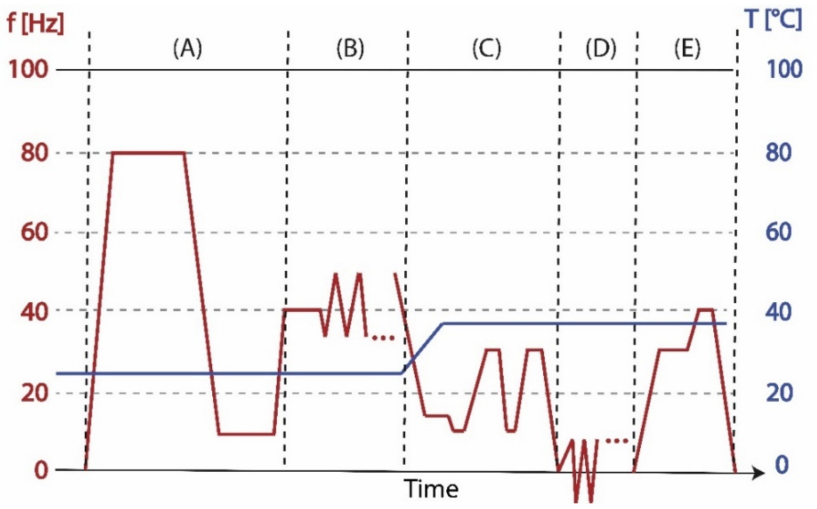

File S1. Overview of the fluidic protocol (schematic) for the automated run of an ImmunoDisk on the LabDisk Player 1, corresponding to Figure 1A in the manuscript. (A) Opening of the stickpack, metering of the assay buffer and inward pumping. Meanwhile, the sample is transferred into the chamber with the fluorescent beads (not yet resuspending them). (B) Loading of the fluorescent beads chamber with the assay buffer and mixing with the sample that had already been transferred there in the previous step. Shake mode mixing to support homogeneous resuspension. Activation of the valve to transport assay buffer, fluorescent beads and sample mix to the multipurpose chamber. (C) Heating to 37 °C. Resuspension of magnetic beads. (D) Incubation with shake mode mixing. (E) Sedimentation of the magnetic beads. Stop at specific position for detection of signal. The protocol parameters are shown in the table below.

Step	Temperature (°C)	Frequency (Hz)	Acceleration (Hz/s)	Duration (s)
1	28			
2		−80	5	60
3		−7	10	20
4		−40	5	20
5		−35	10	0
6		−50	10	0
Loop 5× (step 5–6)				
8		−14	5	2
9	37			5
10		−14	10	1
11		−10	20	10
12		−30	10	20
Loop 1× (step 11–12)				

13		−10	10	5
14		0	10	5
15 (shake mode)		1.5	60	900
16		−30	10	30
17		−40	10	10
18		−4	5	
19 (detection)				

File S2. Sedimentation of magnetic beads

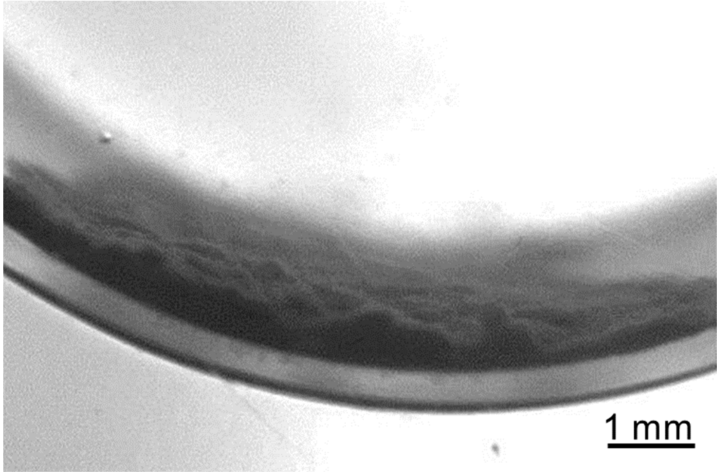

File S2. Magnetic beads in the multipurpose chamber after sedimentation.

File S3. Calculation of the distance traveled by a bead at specific frequencies

Equation for the sedimentation of beads in a centrifugal system by Jacobsen and Sullivan (DOI: 10.1021/i560154a007). The parameters are explained below.

$$r = \sqrt{\frac{18\eta * \ln(\frac{R_{final}}{R_0})}{(\rho_B - \rho_l) * \omega^2 * t}}$$

File S3. The values of the parameters related to our system are shown below (using a 'worst-case scenario' with the maximal distance and a single magnetic bead, without any bound proteins or fluorescent beads on its surface).

Abbreviation	Parameter	Magnetic beads	Fluorescent beads	Others
r	Bead radius	1.4 µm	0.1 µm	
η	Liquid viscosity			1 mPa*s
R_{final}	Final radius of rotation			6.2 cm
R_f	Radius after rotation for a specific time			
R_0	Initial radius of rotation			5.7 cm
ϱ_b	Bead density	1.4 g/ml	1.05 g/ml	
ϱ_l	Density of fluid			0.997 g/ml
ω	Rotational velocity			Variable
t	Time required for sedimentation			Variable

The equation solved for R_{final}:

$$R_{final} = e^{\frac{r^2 * (\rho_b - \rho_l) * \omega^2 * t}{18\eta}} * R_0$$

First step: 30 Hz for 30 s

R_{f1} = 6.06 cm

Second step ($R_0 = R_{f1}$): 40 Hz for 10 s

R_{final} = 6.33 cm

File S4. Results of parametric study for the drying of magnetic beads

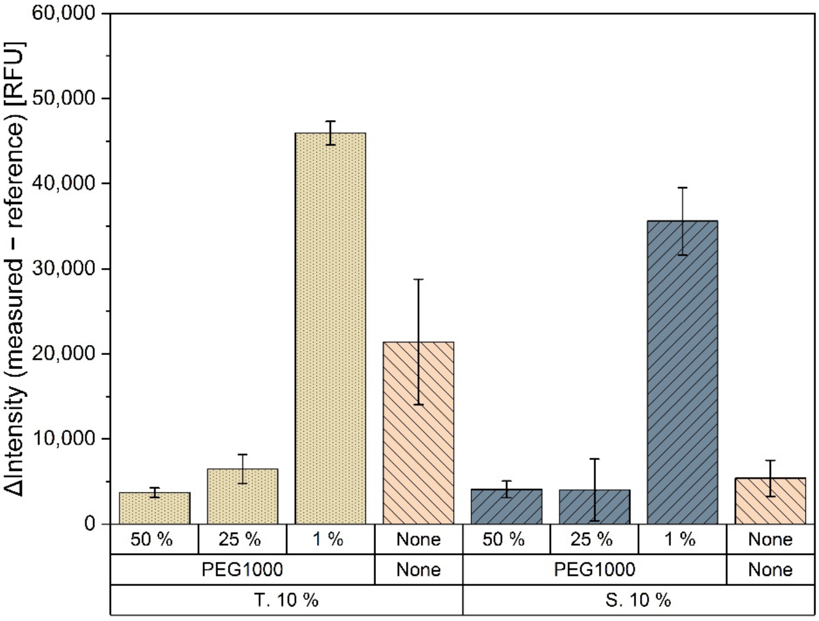

(A) PEG1000 as an additive to trehalose 10% (*w*/*v*) and sucrose 10% (*w*/*v*). All buffers were tested N = 3 except the trehalose 10% (*w*/*v*)/PEG1000 50% (*w*/*v*) and sucrose 10% (*w*/*v*)/PEG1000 50% (*w*/*v*) that were tested N = 10.

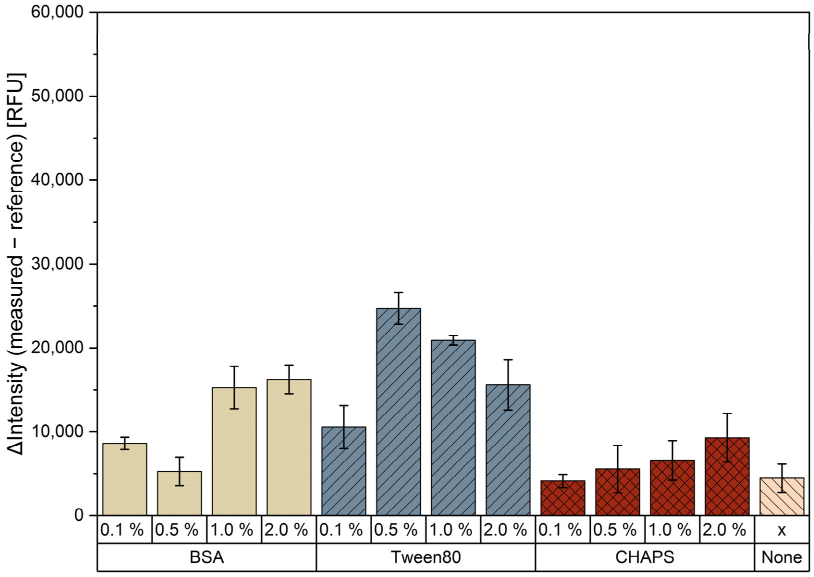

(B) Sucrose 25% (*w*/*v*) with different additives (N = 3).

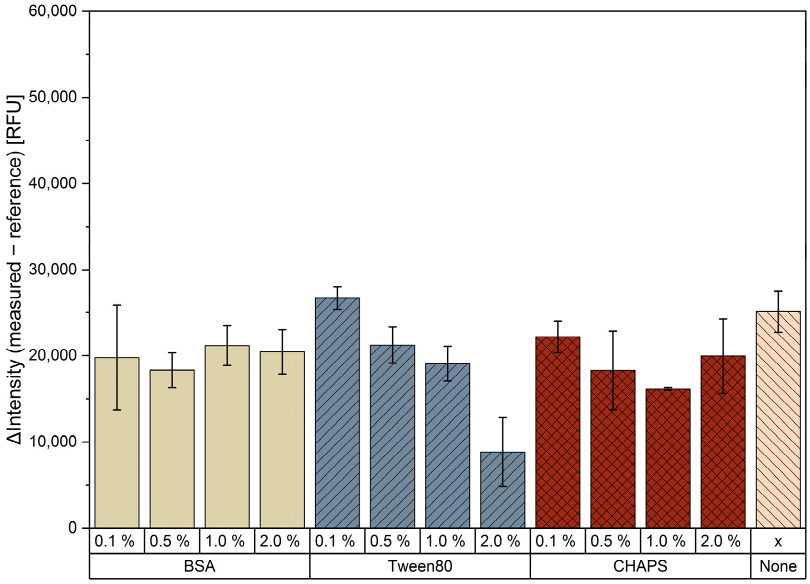

(C) Trehalose 25% (*w*/*v*) with different additives (N = 3).

File S4. ΔIntensity results with different drying buffers used for air drying of magnetic beads coupled with anti-CRP antibodies.

File S5. Magnetic bead resuspension experiments on disk

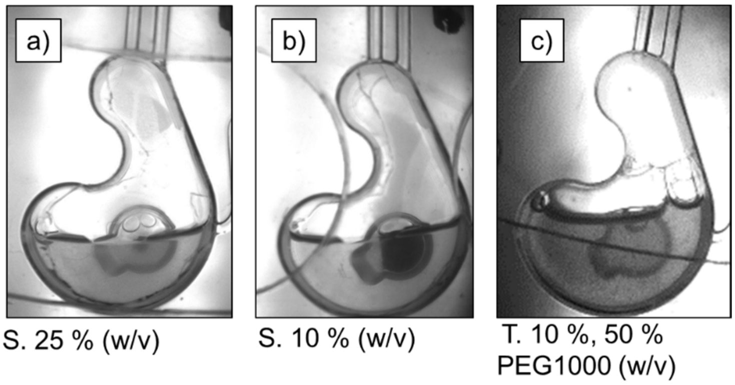

File S5. Exemplary resuspension results of air-dried antibody-coupled magnetic beads on disk with different drying buffers. The drying buffers (a) and (c) were the best-performing among those shown in the manuscript Figure 3B. The image (b) shows an example of a drying buffer that did not perform well when the magnetic beads were dried in the microtiter plate and also leads to incomplete resuspension results on disk.

File S6. Overview of different drying buffers for native CRP-coupled fluorescent beads

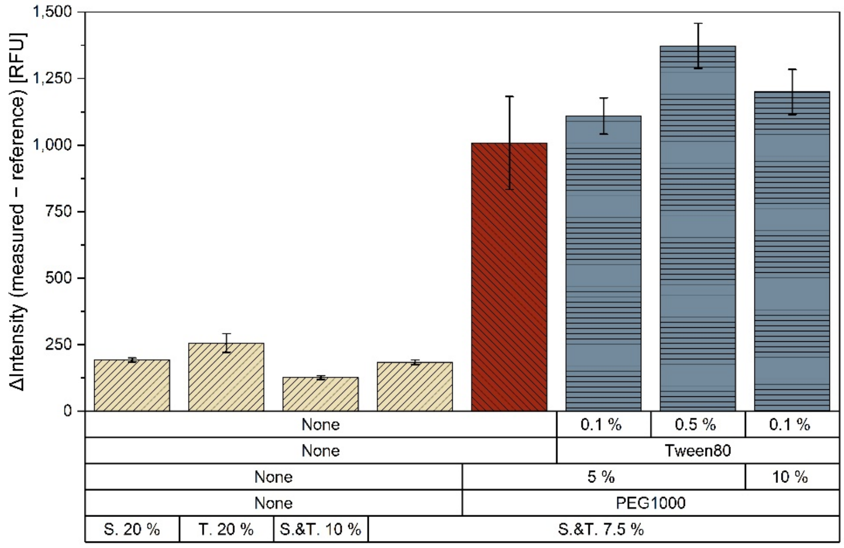

File S6. Other drying buffers (including diverse additives) for air drying of CRP antigen-coupled fluorescent beads (N = 3) compared to the drying buffers with no additives (also shown in Figure 3C).

File S7. MMP-9 assay with dried antibody-coated magnetic beads and liquid antigen-coated fluorescent beads

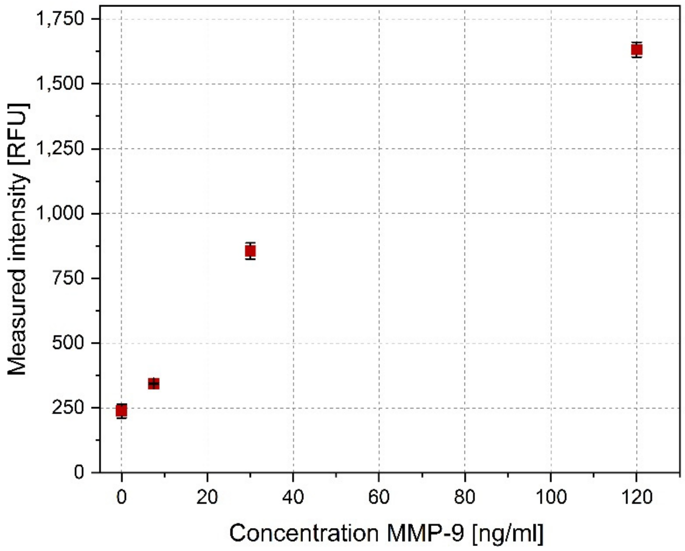

File S7. Few selected concentrations (N = 2) performed on disk for a feasibility test of the drying of magnetic beads coupled with antibodies against human matrix metalloproteinase 9 (MMP-9).

File S8. Design of Experiments (DoE)

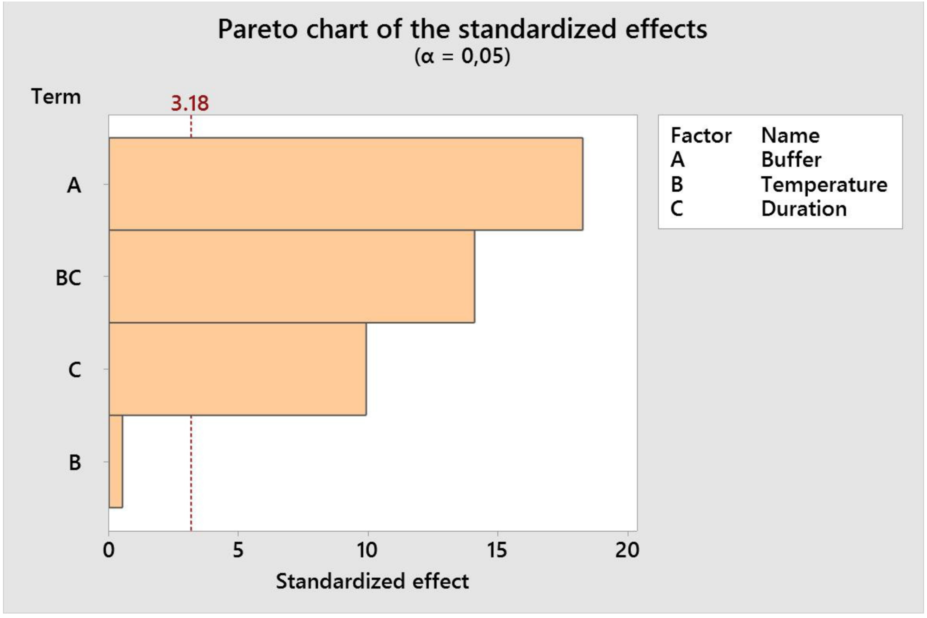

File S8. Pareto chart of the DoE conducted to investigate the influence of drying buffer, drying temperature and drying duration on the assay result after both protein-coupled magnetic and fluorescent beads were dried with different parameters.

Overview of parameters investigated with the DoE:

- Drying buffer for antibody-coupled magnetic beads (unchanged): PBS with 50% (*w*/*v*) PEG1000 and 10% (*w*/*v*) trehalose
- Drying buffer for antigen-coupled fluorescent beads:
    - PBS with 10% (*w*/*v*) trehalose and 10% (*w*/*v*) sucrose
    - PBS with 10% (*w*/*v*) trehalose, 10% (*w*/*v*) sucrose and 5% (*w*/*v*) PEG1000
- Drying temperature:
    - High: 45 °C
    - Low: 37 °C
- Drying durations:
    - Long: 3 h
    - Short: 0.5 h

File S9. Overview of ΔIntensity and CV using different drying temperatures and durations

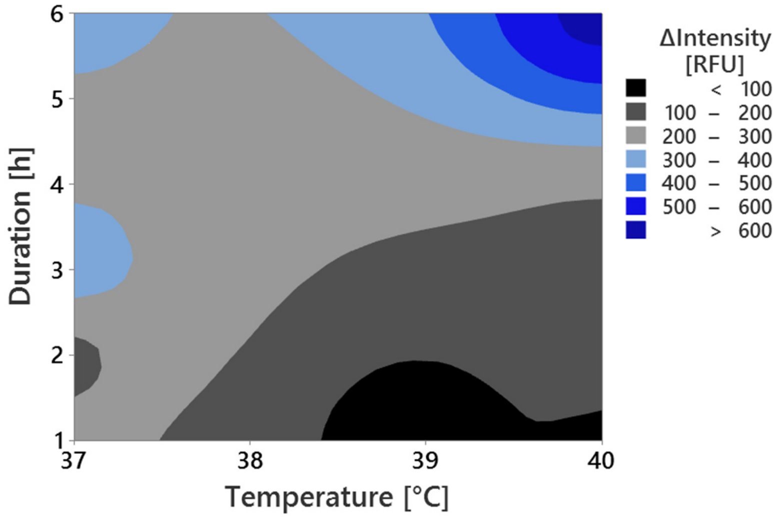

File S9.1. Study of different drying temperatures and durations using the final drying buffer for MB (trehalose 10% (*w*/*v*), PEG1000 50% (*w*/*v*) in PBS) and FB (trehalose and sucrose 10% (*w*/*v*) in PBS) on disk evaluating the ΔIntensity of the measured signal versus the non-dried reference assay.

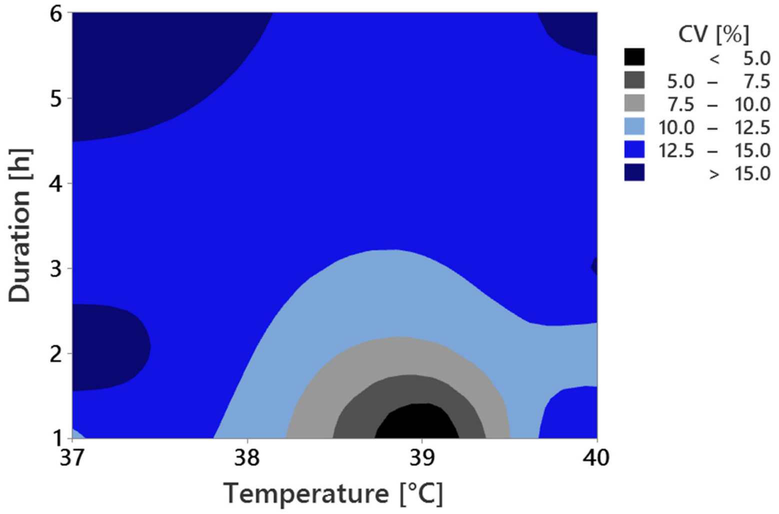

File S9.2. Study of different drying temperatures and durations using the final drying buffer for MB (trehalose 10% (*w*/*v*), PEG1000 50% (*w*/*v*) in PBS) and FB (trehalose and sucrose 10% (*w*/*v*) in PBS) on disk evaluating the CV of the measured signal versus the non-dried reference assay.

File S10. Calibration curve

Sigmoidal fit curve:

$$x = x_0 \sqrt[p]{\frac{y - A_1}{A_2 - y}}$$

Parameter	Value of calibration curve
**A1**	155.490
**A2**	1445.050
**x0**	67.981
**p**	2.349

Equation for the calculation of LOB and LOD:

$$LOB = Intensity_{blank} + (1.645 * standard\ \ deviation_{blank})$$

$$LOD = LOB + (1.645 * standard\ \ deviation_{lowestconc.})$$

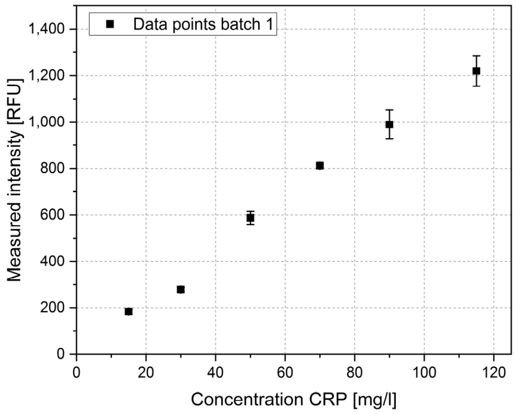

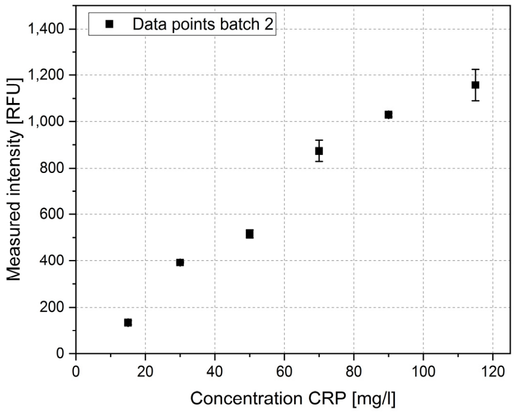

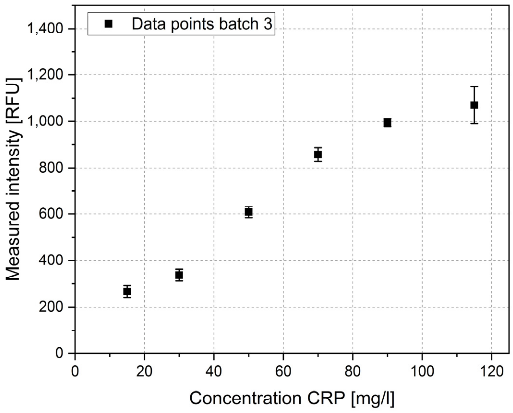

File S10. Calibration curves of batches 1, 2 and 3 normalized to their respective blank that were used to calculate the overall calibration curve shown in manuscript Figure 4A.

File S11. Data of CRM measurements and outlier calculation

	Batch 1	Batch 2	Batch 3
	**Calculated CRP concentration (mg/l)**		
	42.3	50.7	42.2
	53.8	47.9	43.3
	49.6	Outlier	49.5
	43.6	43.6	49.6
Average (mg/l)	47.3	47.4	46.1
Stdev (mg/l)	5.3	3.6	3.9
CV (%)	11.3	7.6	8.6

Box chart calculation to evaluate outlier:

Q1 = 43.41 mg/l
IQR = 5.86
Q3 = 49.27 mg/l
Lower range limit: 34.6 mg/l
Upper range limit: 58.1 mg/l
Outlier: 64.1 mg/l
